# A Mechanical Defect Localization and Identification Method for High-Voltage Circuit Breakers Based on the Segmentation of Vibration Signals and Extraction of Chaotic Features

**DOI:** 10.3390/s23167201

**Published:** 2023-08-16

**Authors:** Shi Cao, Tong Zhao, Gang Wang, Tigui Zhang, Chenlei Liu, Qinzhe Liu, Zhenming Zhang, Xiaolong Wang

**Affiliations:** 1 School of Electrical Engineering, Shandong University, Jinan 250061, China; caoshi@mail.sdu.edu.cn (S.C.); liu_cl@sdu.edu.cn (C.L.); qinzhe111@163.com (Q.L.); zhangzhenm1998@163.com (Z.Z.); wangxiaolong@sdu.edu.cn (X.W.); 2 Shandong Taikai Automation Co., Ltd., Tai’an 271000, China; wangg0312@163.com; 3 Shandong Taikai Disconnector Co., Ltd., Tai’an 271000, China; tkglqyfzb@163.com

**Keywords:** high-voltage circuit breaker, mechanical defect, vibration signal, phase space reconstruction, chaotic features

## Abstract

To address the problem of mechanical defect identification in a high-voltage circuit breaker (HVCB), this paper studies the circuit breaker vibration signal and proposes a method of feature extraction based on phase-space reconstruction of the vibration substages. To locate mechanical defects in circuit breakers, vibration signals are divided into different substages according to the time sequence of the parts of the circuit breakers. The largest Lyapunov exponent (LLE) of the vibration signals’ substages is calculated, and then the substages are reconstructed in high-dimensional phase space. The geometric features of the phase trajectory mean center distance (MCD) and vector diameter offset (VDO) are calculated, and the LLE, MCD, and VDO are selected as the three fault identification features of the vibration substages. The eigenvalue anomaly rate of each substage of the vibration signal under defect state are calculated and analyzed to locate the vibration substage of the mechanical defect. Finally, a fault diagnosis model is constructed by a support vector machine (SVM), and the common mechanical defects of circuit breakers simulated in the laboratory are effectively identified.

## 1. Introduction

High-voltage circuit breakers (HVCBs) are very important pieces of equipment used for control and protection in the power system. They can disconnect the current and protect other equipment in the power grid. The faulty operation of a circuit breaker can seriously affect the safety and stability of the power system [1,2,3,4]. According to an international investigation [5], approximately 50% of circuit breaker failures are caused by mechanical failure, and operating mechanism failure is the main source of circuit breaker mechanical failure. The early stage of mechanical failure often only consists of a small mechanical defect, which does not affect the normal operation of the circuit breaker and is not easy to detect. However, if it is not found in time, the mechanical defect will evolve into a mechanical fault, which seriously affects the safe and stable operation of the circuit breaker [6,7].

During the operation of a circuit breaker, the vibration signal generated by the collisions between the parts contains rich mechanical status information, which can be used to detect mechanical faults. In recent years, research on HVCB fault diagnosis based on vibration signals has become a focus of scholars [8,9,10,11,12,13,14,15,16,17]. In the field of fault diagnosis, in addition to single variable fault diagnosis, multivariate statistical analysis is also an important research tool which has been widely applied for fault diagnosis of electrical systems [18,19,20,21]. The traditional time–frequency analysis method of vibration signals has many shortcomings; for example, the wavelet transform has some problems, such as basis function selection and energy leakage, and it is not self-adaptable. Empirical mode decomposition (EMD) also has some problems, such as mode aliasing and end effects. A new adaptive signal decomposition method proposed by Dragomiretskiy and Zosso, namely, variational mode decomposition (VMD) [22], overcomes the shortcomings of traditional time–frequency analysis methods to a large extent, but it is still a linear system analysis method in essence.

As a physical system with a complex structure, the circuit breaker has many internal mechanical components, and there are gaps, friction, flexibility differences, and inconsistent thermodynamic parameters between different components. Thus, the behavior of the system is strongly nonlinear. The signal analysis method based on traditional linear time series cannot be effectively analyzed, but nonlinear time series analysis methods (such as the phase-space reconstruction technology of chaos theory) are more suitable to describe the irregularities of such systems. Ergodic theory [23] holds that one-dimensional time series contain very rich system information and trajectories of all variables involved in dynamic system evolution. Phase-space reconstruction of time series is an effective method to extract rich variable information from univariate time series. The method of phase-space reconstruction is used to analyze nonlinear systems and then realize fault diagnosis, which has been applied in the field of circuit breaker mechanical fault diagnosis [24,25,26,27]. Wu et al. [24] were the first to use phase-space reconstruction technology to process circuit breaker vibration signals and extract their correlations to evaluate the mechanical states of circuit breakers. Yang et al. [27] extracted the chaotic feature quantity largest Lyapunov exponent, correlation dimension and Kolmogorov entropy as a set of features for circuit breaker state recognition based on chaotic dynamics theory and achieved good recognition results. The above research shows that phase-space reconstruction has been widely used in the field of circuit breaker mechanical fault diagnosis and has produced good recognition results. However, current research is aimed at the whole vibration signal during the opening and closing process of the circuit breaker. There is a lack of specific analysis of the operation process of the circuit breaker and research on the characteristics of the vibration signals generated in different operation substages. It is difficult to realize the localization and identification of early mechanical defects of the circuit breaker operating mechanism.

To solve the problem that the mechanical defects of circuit breakers cannot be found accurately and in a timely manner, this paper proposes a method to extract the chaotic features of the vibration signal substages and calculate the eigenvalue anomaly rate to locate and identify mechanical defects. First, the vibration signals are divided into different vibration substages according to the operation characteristics of different circuit breaker types. The largest Lyapunov exponent is calculated for the vibration signal of each substage, and phase-space reconstruction is used to reconstruct the attractor and then extract the morphological features of the attractor. The largest Lyapunov exponent and attractor features are used as signal fault characteristic values to distinguish the different operating states of the circuit breakers. The eigenvalue anomaly rate of each vibration signal substage is calculated, and the calculated results are compared with the threshold value of the abnormal rate to locate the abnormal signal in the vibration substages. Finally, the features of each vibration substage of the vibration signal compose the feature vector, and the fault diagnosis model is constructed by SVM to realize the accurate identification of different types of mechanical defects.

## 2. Chaotic Feature Extraction Method of Vibration Signals Based on Phase-Space Reconstruction

### 2.1. Phase-Space Reconstruction

The HVCB is a complex nonlinear dynamic system, and the vibration signals generated during its operation are a complex nonlinear time series, which contains rich information about the circuit breaker system. This information can be extracted by phase-space reconstruction technology. Packard et al. [28] proposed reconstructing the phase space through the delay coordinates of variables to restore the dynamic characteristics of the system. For a given time series {x(i),i=1,2,…,N}, where *N* is the length of the time series, its reconstructed phase-space coordinates can be expressed as:

(1) $$\left\{\begin{array}{c} X(1)=\{x(1),x(1+\tau ),\ldots ,x(1+(m-1)\tau )\}\\ X(2)=\{x(2),x(2+\tau ),\ldots ,x(2+(m-1)\tau )\}\\ \ldots \\ X(i)=\{x(i),x(i+\tau ),\ldots ,x(i+(m-1)\tau )\} \end{array}\right.$$

where X(i) are the phase point coordinates in the reconstructed phase space, x(i) are the sample point values of time series, τ is the time delay, and m is the embedding dimension. The key to phase-space reconstruction lies in the determination of the time delay and embedding dimension [29].

#### 2.1.1. Time Delay

In this paper, the mutual information method [30] is chosen to determine the time delay, and the principle is as follows:

Assuming that the original vibration signal S={x(i),i=1,2,…,N} becomes Q={x(i+τ),i=1,2,…,N} after the time delay τ, the mutual information value between the sequence *S* and *Q* can be obtained by the information entropy and probability as follows:

(2) $$\begin{array}{l} I(Q,S)=H(S)+H(Q)-H(S,Q)\\ =-\sum_{i}P_{s}(s_{i})\log P_{s}(s_{i})-\sum_{j}P_{q}(q_{j})\log P_{q}(q_{j})\\ +\sum_{i}\sum_{j}P_{sq}(s_{i},q_{j})\log[\frac{P_{sq}(s_{i},q_{j})}{P_{s}(s_{i})P_{q}(q_{j})}] \end{array}$$

where H(S) and H(Q) are the information entropy of signals *S* and *Q*, respectively; H(S,Q) is the joint information entropy of *S* and *Q*, respectively; $P_{s}(s_{i})$ is the probability of the point with the median sampling point being $s_{i}$ appearing in the entire data segment; $P_{q}(q_{j})$ is the probability of the point with the median sampling point being $q_{j}$ appearing in the entire data segment; and $P_{sq}(s_{i},q_{j})$ is the probability that the original signal has a sampling value of $s_{i}$ and the delayed signal has a sampling value of $q_{j}$. According to the curve of mutual information versus delay time, the delay time corresponding to the first local minimum of the curve is the best time delay for phase-space reconstruction [30].

**Figure 1:**
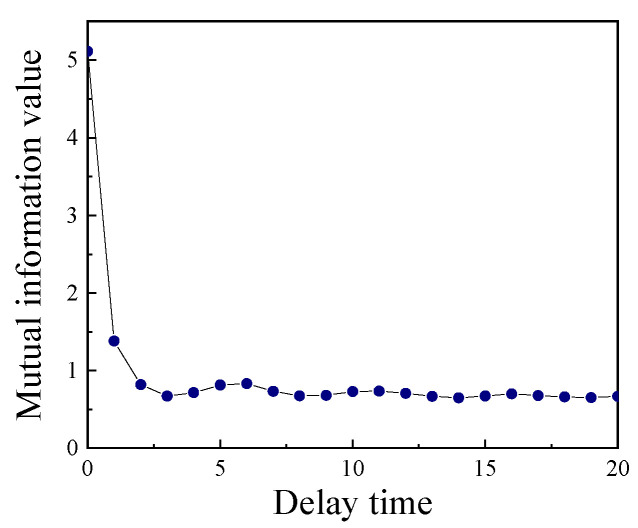
Mutual information value curve changing with time delay.

The mutual information curve drawn according to a typical vibration signal of the circuit breaker is shown in Figure 1. According to the curve, the first local minimum appears at τ=3, so the best time delay is τ=3.

#### 2.1.2. Embedding Dimension

In this paper, the false nearest neighbor (FNN) method is used to determine the optimal embedding dimension. The principle of the FNN method is as follows [31]: when the embedding dimension is very small, the trajectory in phase space is not fully developed, and the trajectory is wound and folded together, making some points that were originally far away become very close; these near points are called false nearest points. With the increase in the embedding dimension, the phase trajectory gradually expands, making the compressed false neighbors gradually separate so that they are no longer adjacent phase points. The embedding dimension when all the false neighbors disappear is the optimal embedding dimension.

**Figure 2:**
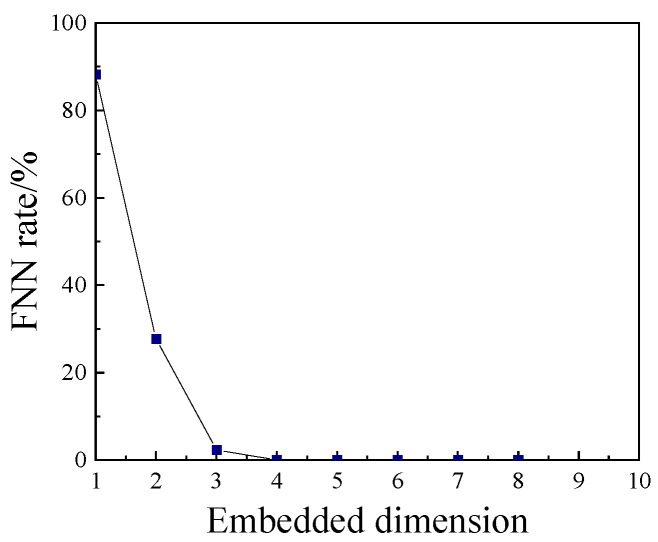
FNN rate with embedding dimension.

According to the typical vibration signal of the circuit breaker, the curve of the FNN rate with the embedding dimension is drawn in Figure 2. According to the curve, when the embedding dimension reaches 3, the FNN rate has basically decreased to 0, so the embedded dimension is chosen as m=3.

### 2.2. Chaotic Features of the Vibration Signal

#### 2.2.1. Largest Lyapunov Exponent

The phase-space reconstruction technique is the most common method for dealing with chaotic time series. Before the phase-space reconstruction of the signal, it should first be proven that the signal is chaotic [32]. In this paper, the largest Lyapunov exponent (LLE) is selected as the criterion for judging the chaos of circuit breaker vibration signals. If the largest Lyapunov exponent is positive, the signal is chaotic, and the larger the LLE value is, the stronger the chaos of the signal [33].

In this paper, the Wolf algorithm is used to calculate LLE. The calculation steps are as follows:

1. First, the initial vector is constructed, the initial point $x_{0}$ corresponding to the initial time $t_{0}$ is selected in the time series, and the phase locus point $x_{j}$, which is closest to $x_{0}$, is selected in the high-dimensional phase space to form the initial vector. The Euclidean distance between the two phase points is calculated as $L(t_{0})$;
2. Assume that the time step is k. After a time step, $t_{1}=t_{0}+k$, a new vector is obtained, and the Euclidean distance $L(t_{1})$ between the base point $x_{1}$ and the endpoint is calculated. In the corresponding time step, the exponential growth rate of the phase point distance is $\lambda_{1}=\frac{1}{k}\log_{2}\frac{L(t_{1})}{L(t_{0})}$;
3. The estimated value of LLE is obtained by summing the growth rate of each linear index of the system and taking the average value: $\mathrm{LLE}=\frac{1}{N}\sum_{i=1}^{N}\frac{1}{k}\log_{2}\frac{L(t_{i})}{L(t_{i-1})}$, where *N* is the number of phase points;
4. While increasing the embedding dimension m, repeat the above steps until LLE changes smoothly; the resulting value is the largest Lyapunov exponent.

The largest Lyapunov exponent can be used not only as a discriminator of system chaos, but also to reflect the mechanical state of the circuit breaker [34]. As an output of a complex nonlinear dynamic system, the vibration signal of a circuit breaker is generated by the interaction of various mechanical parts. The chaotic degree of the vibration signal can reflect the health state of the mechanical parts of the operating mechanism. The largest Lyapunov exponent is an important index to judge the strength of the chaos of the system, and it is sensitive to changes in the motion state of the dynamic system. It can quantitatively describe the chaotic properties of the system, so this paper uses LLE as the fault-sensitive feature to identify the mechanical defects of circuit breakers.

#### 2.2.2. Phase Trajectory Feature

Phase-space reconstruction technology can map one-dimensional time series of vibration signals to high-dimensional phase space to recover all state information of a dynamic system. In a complex dynamic system, any small mechanical defect leads to a change in the geometric shape of the chaotic attractor in high-dimensional space, i.e., the space of the vibration signal of the circuit breaker. The morphological features of the attractor can reflect the change in the mechanical state of the circuit breaker [35], and the geometric features of the attractor can be used as the characteristic value of the mechanical state of the circuit breaker. The morphological features of the attractor are also very sensitive to the operating states of the circuit breaker, and the changes in the operating state of the circuit breaker can be reflected by extracting the morphological features of the attractor. The mean center distance and vector diameter offset can give a good description of the morphological changes in the attractor. In this paper, the mean center distance and vector diameter offset are introduced to describe the geometric form of the attractor in phase space to recognize the common mechanical defects of circuit breakers.

Mean of center distance (MCD): After phase-space reconstruction, the shape of the chaotic attractor of the vibration signal is approximately a hypersphere in phase space, and the origin of the phase-space coordinates can be considered as approximately the center of the hypersphere. To quantify the degree of aggregation of chaotic attractors toward the center of the sphere, the mean of the sum of distances between all vector points in phase space and the origin of the phase-space coordinates is defined as the mean of the center distance. The formula is as follows:

(3) $$S=\frac{1}{N}\sum_{i=1}^{N}{\sqrt{x{\left(i\right)}^{2}+x{\left(i+\tau \right)}^{2}+\ldots +x{\left(i+(m-1)*\tau \right)}^{2}}}^{}$$

where (x(i),x(i+τ)…x(i+(m−1)*τ)) are the coordinates of the points in phase space, *N* is the number of state points, and m is the embedding dimension.

Vector diameter offset (VDO): The two-dimensional phase trajectory is an approximate circle, and it is distributed on both sides of the diagonal of the first and third quadrants of the two-dimensional coordinate axis. Extending to the high-dimensional space, the chaotic attractors of vibration signals are distributed on both sides of the main diagonal of the high-dimensional space coordinate system. In this paper, the vector diameter offset (relative to the main diagonal direction) is selected to quantify this geometric characteristic of the attractor. Geometrically, the vector diameter offset is the distance between the state point and the main diagonal, and the calculation formula is as follows:

(4) $$L=\frac{1}{N}\times \sum_{i=1}^{N}r_{i}\sin\delta_{i}=\frac{1}{N}\times \sum_{i=1}^{N}\frac{{\left\|X(i)\right\|}_{2}}{\sqrt{m}}$$

where *N* is the number of state points, $r_{i}$ is the vector diameter of the state points, $\delta_{i}$ is the angle between the state vector and the main diagonal, X(i) is the state vector in high-dimensional space, and m is the embedding dimension.

## 3. A Time-Sharing Division Processing Method for Vibration Signals during Circuit Breaker Operation

The vibration signal of the HVCB is the result of the joint action of many mechanical components. Although its duration is very short, it contains rich event information. The vibration signal of the circuit breaker is composed of multiple vibration substages. In many cases, a mechanical defect of the circuit breaker only introduces abnormality in the corresponding vibration substage and has little influence on other vibration substages. At this time, if the whole vibration signal is analyzed to extract the fault feature, the anomaly caused by the mechanical defect will be submerged in the overall vibration signal, and identifying the defect is difficult. To accurately locate mechanical defects and improve the sensitivity of fault feature identification of mechanical defects, this paper first processed the vibration signals by time-sharing division according to the action timing of the parts of the circuit breaker operating mechanism, analyzed the parts’ action events corresponding to each substage, and then reconstructed the signals of different vibration substages in phase space. Finally, the chaotic features of different vibration substages were extracted.

### 3.1. Time-Sharing Division of Vibration Signals

**Figure 3:**
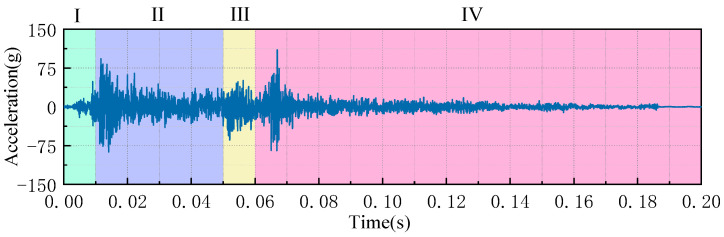
The switching vibration signal is divided into different substages.

Figure 3 shows the waveform diagram of a typical opening vibration signal of an HVCB spring operating mechanism. The vibration signal contains multiple vibration substages. According to the action process of the circuit breaker operating mechanism (tripping trigger, spring release, mechanism transmission, brake limit, etc.), the opening vibration signal is divided into the following substages.

Substage I is the pawl trip stage (0~10 ms), which includes the event where the circuit breaker receives the opening command, the opening electromagnet moves the core to draw, and it drives the opening guide rod to impact the opening switch;

Substage II is the spring release stage (10~50 ms), which is the stage after the switch release, the consumption of energy of the switch spring release, and the mechanism solution column release;

Substage III is the mechanism transmission stage (50~60 ms), and the energy released by the spring makes the spindle drive shaft rotate through the crank arm, driving the breaker contact to separate;

Substage IV is the mechanism braking stage (60~200 ms). In this stage, the contact terminal moves to the lowest position. Under the joint action of the buffer and the opening spring, the contact terminal gradually recovers, and the opening operation is completed.

The mechanical structure characteristics and operation principle of the spring-operated mechanism type circuit breaker are very similar, and the vibration signal can be divided into substages according to the above segmentation method.

### 3.2. Vibration Signal Phase-Space Reconstruction in Substages

**Figure 4:**
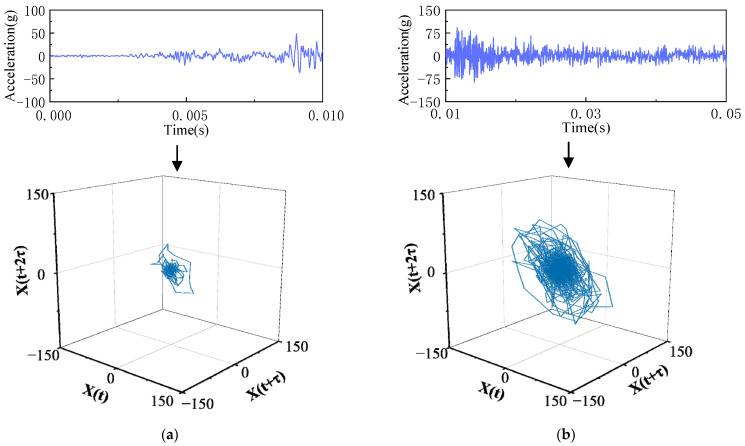

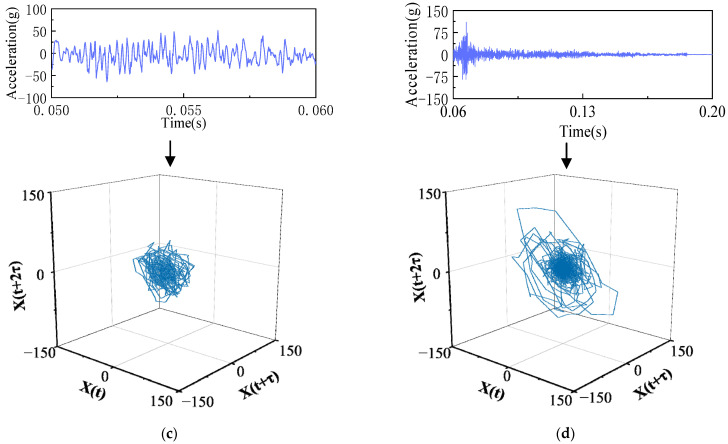
Phase-space reconstruction results of the vibration signal at different substages. (**a**) Vibrational substage I. (**b**) Vibrational substage II. (**c**) Vibrational substage III. (**d**) Vibrational substage IV.

The phase-space reconstruction technique is used to reconstruct the vibration signals of each substage to the high-dimensional phase space after the time-sharing division processing of circuit breaker vibration signals. As shown in Figure 4, the time-domain waveform and phase-space track diagram of vibration signal substages after time-sharing division processing are drawn. The phase-space signals at each vibration substage are obviously different.

One can see by comparing the waveforms and phase-space trajectories of each substage of the vibration signal that the phase trajectories of different substages of the same vibration signal are significantly different, and the geometry of the phase trajectory is related to the time-domain waveform of the vibration signal. When the time-domain waveform impact on the vibration signals is relatively gentle, the phase trajectory distribution is relatively clustered, and the shape is small, but when the time-domain waveform peak impact is more pronounced, the corresponding phase-space trajectory distribution is more dispersed, and the shape is larger. Due to the limitation of the time-domain analysis method, the time-domain waveform of the circuit breaker vibration signal cannot be used to effectively identify all mechanical defects, but the phase trajectory properties of the vibration signal are equivalent to the properties of the original dynamic system, and the mechanical defects of the circuit breaker operating mechanism can be well identified.

**Figure 5:**
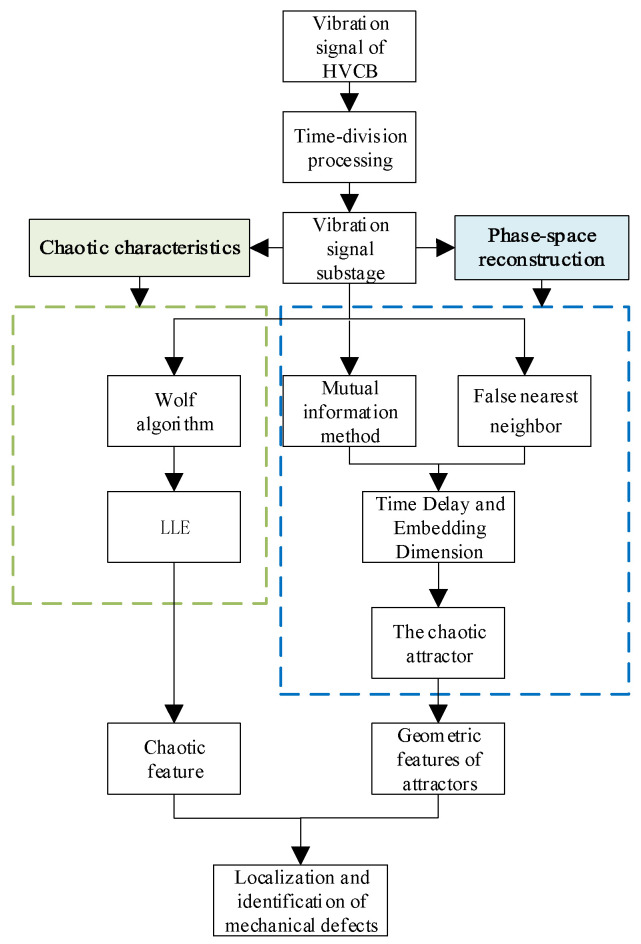
Flow chart of mechanical defect identification.

Based on this, the chaotic features of different substage vibration signals are extracted through phase-space reconstruction, and the rapid location and identification of circuit breaker mechanical defects is realized. First, the Wolf algorithm is used to calculate the LLE of the vibration substage signal to judge its chaotic properties, and the LLE is used as the chaotic characteristic index of mechanical defect identification. Then, the phase-space reconstruction technique is used to reconstruct the corresponding vibration subphase signal in high-dimensional phase space. The MCD and VDO of the phase trajectories are extracted as the phase trajectory geometric feature indexes for defect identification. The LLE, MCD, and VDO of the vibration signals in each substage are taken as characteristic values to calculate the eigenvalue anomaly rate index of the fault signal and normal signal in each substage, and the abnormal signal is located according to the eigenvalue anomaly rate curve. Finally, the eigenvalues of the signals in each substage are composed of characteristic vectors, and the classification and recognition of the different operating states of circuit breakers are realized through SVM. The specific process is shown in Figure 5.

## 4. Vibration Signal Feature Extraction and Mechanical Defect Identification of the Circuit Breaker Operating Mechanism

### 4.1. Mechanical Defect Simulation and Vibration Signal Monitoring of the Circuit Breaker Operating Mechanism

**Figure 6:**
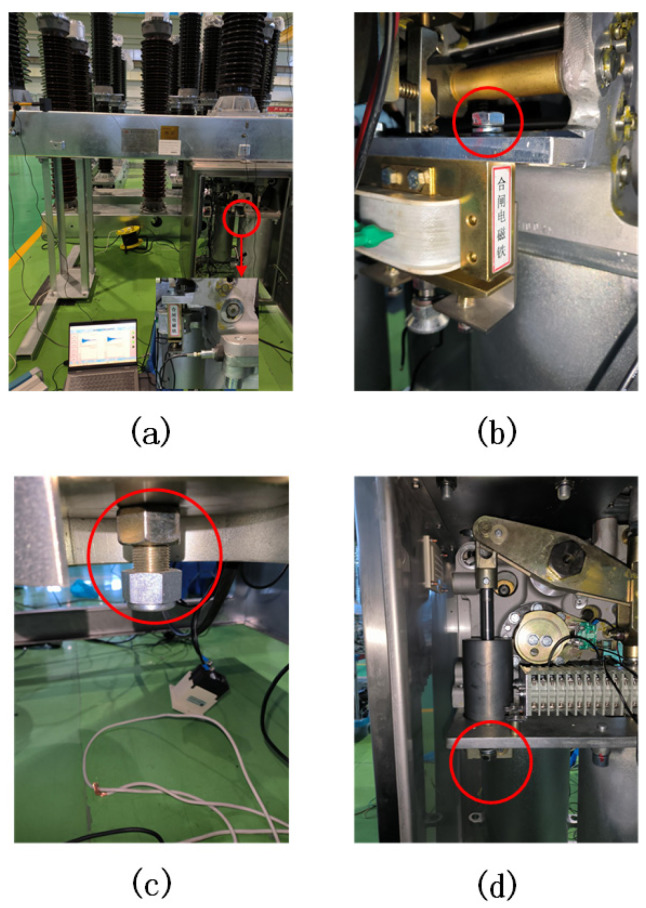
Experimental arrangement and defect simulation. (**a**) Sensor connection. (**b**) Closing electromagnet looseness (the text in the figure means closing electromagnet). (**c**) Operating mechanism jam. (**d**) Shock absorber malfunction.

To verify the method proposed in this paper, the LW30-72.5 high-voltage AC SF_6_ porcelain post circuit breaker is taken as a sample to collect circuit breaker on–off vibration signals, and the test layout is shown in Figure 6a. The high-voltage circuit breaker used in the case operated on a spring mechanism, and a vibration acceleration sensor (type IEPE) is used with a sensitivity of 50 mv/g, a maximum range of 150 g, and a frequency response range of 0.5~10,000 Hz (±10%). An NI-9234 vibration sound data acquisition card was used in the experiment, which has four analog signal inputs with synchronous sampling and 24-bit resolution, and the maximum sampling rate of each channel is 51.2 kS/s. Since the vibration signal of the circuit breaker propagates along the solid material, and the attenuation is small, to obtain the vibration signal that can contain the component information of the circuit breaker, the measurement point should be selected near the vibration propagation path and be convenient for the installation of the sensor. In this experiment, the acceleration sensor is placed on the side of the closing spring sleeve inside the operating mechanism box to enable clearer and more stable opening and closing vibration signals.

This case simulated three common mechanical defects of the circuit breaker operating mechanism: (1) loose closing electromagnet (LCE) (simulated by loosening the fixing screw of the closing electromagnet, as shown in Figure 6b); (2) an operating mechanism jam (OMJ) (reduces the precompression value of the opening spring by adjusting the pretightening screw of the opening spring to simulate an opening jam defect, as shown in Figure 6c); and (3) shock absorber malfunction (SAM) (simulates the malfunction of the shock absorber by loosening the fastening screws of the oil absorber, as shown in Figure 6d). In this case, 15 groups of data were collected for each type of vibration signal under normal closing (NC), normal opening (NO), and the three defect states.

### 4.2. Chaotic Feature Extraction of the Vibration Signal Substage

#### 4.2.1. LLE of the Vibration Signal Substage

To extract the chaotic features of the vibrator signals of the HVCB under different operating states, the Wolf algorithm is used to calculate the LLE of each vibration substage; the calculation results are shown in Table 1. The LLE of each vibration substage under the different operating states is positive, which proves that the vibration signals of different substages under different operating states are chaotic and can be analyzed by the phase-space reconstruction method. At the same time, the LLEs of the circuit breaker in different substages under the same operating state are significantly different, and the LLEs of the same vibration substage under different operating states are also significantly different. Therefore, the LLE can be used as a fault-sensitive feature to identify the different mechanical states of the circuit breaker.

#### 4.2.2. Trajectory Features of the Vibration Signal Substage

**Figure 7:**
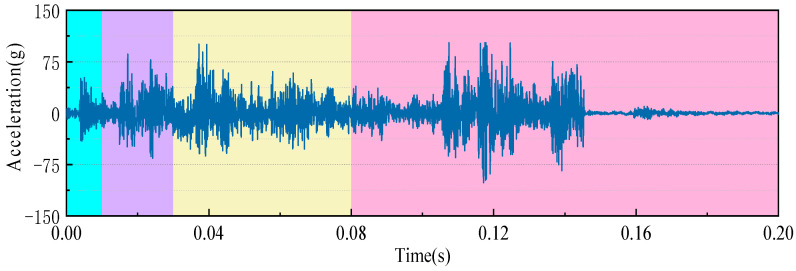
Different substages of the closing vibration signal.

To accurately locate abnormal signals and improve the detection sensitivity of mechanical defects, time-sharing division processing is performed before phase-space reconstruction [36]. The closing vibration signal of the circuit breaker is divided into four substages (tripping substage, spring release substage, transmission substage, and brake substage) according to the above time-sharing division processing method. The result of time sharing and segmentation processing is shown in Figure 7. As a result of the different parts required by the opening and closing actions, the times of the vibration subevents are not exactly the same, so the range of the vibration substages after the segmentation of the closing vibration signal is different from that of the opening vibration signal.

**Figure 8:**
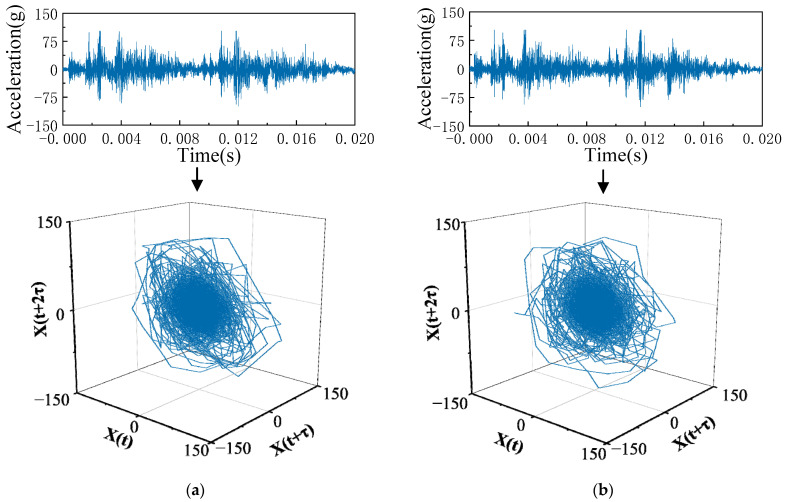
Results of the phase-space reconstruction of the vibration signal under normal and defect closing. (**a**) NC state. (**b**) LCE state.

The vibration signals of NC and LCE are shown in Figure 8. The result of the phase-space reconstruction of the two vibration signals shows that it is difficult to identify the fault operating state of the circuit breaker from the waveform of the vibration signal or the phase-space track.

**Figure 9:**
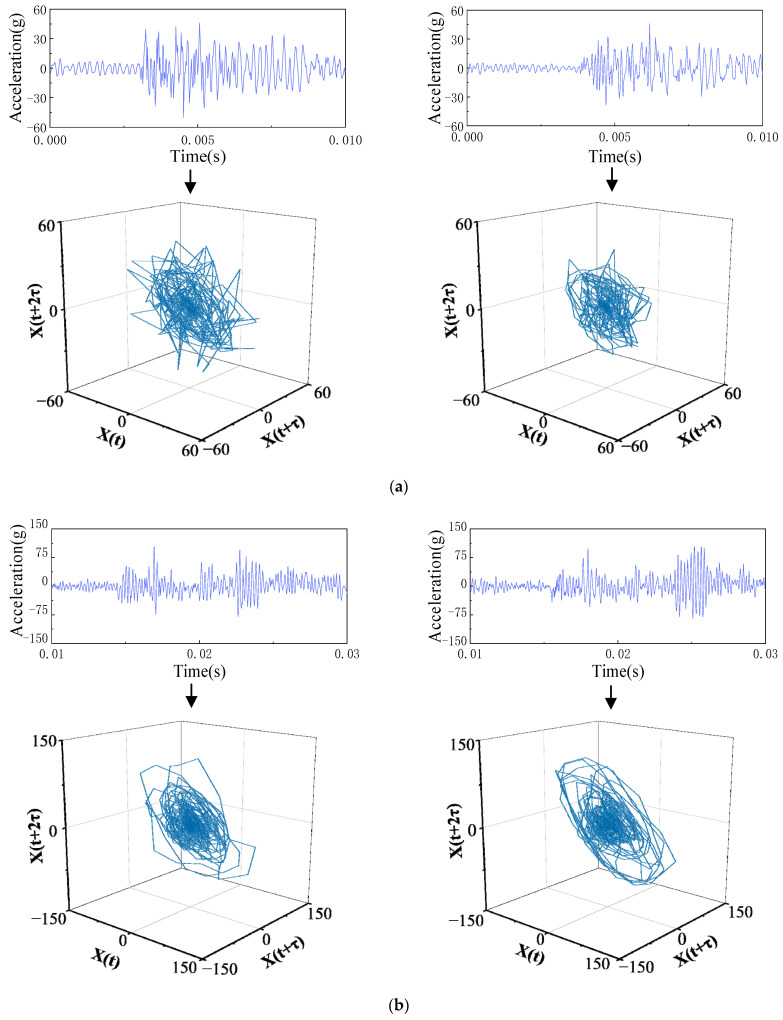

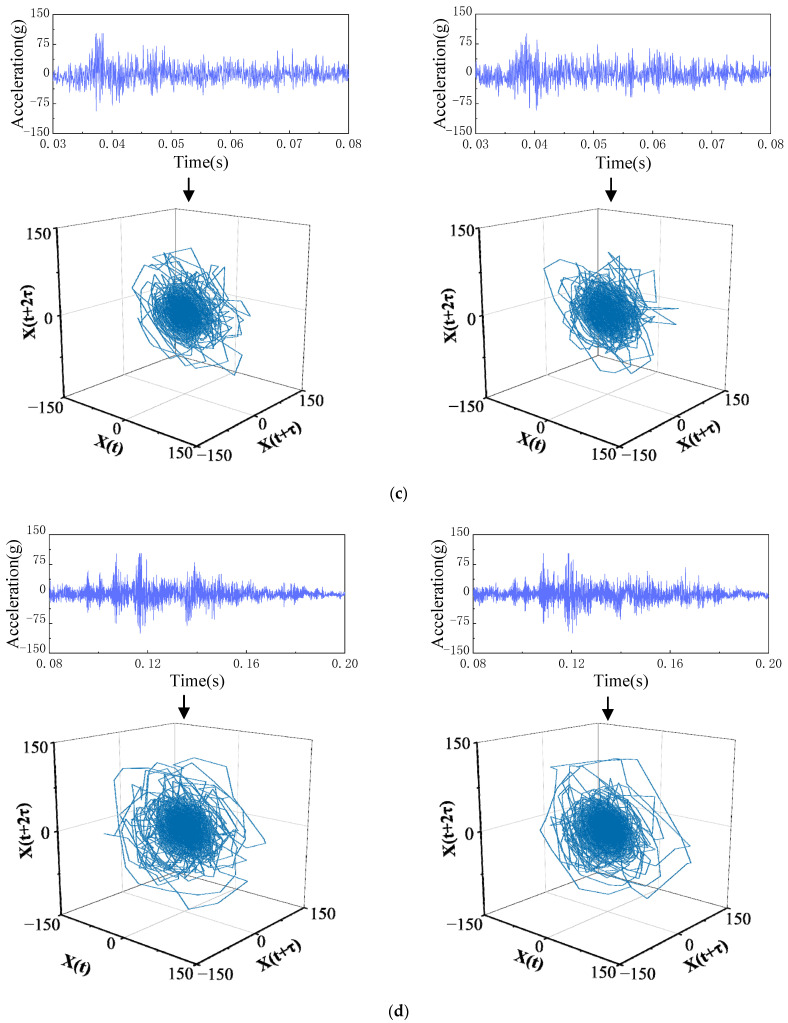
Phase-space reconstruction results of each vibration substage of normal and defect closing. (**a**) NC I and LCE I. (**b**) NC II and LCE II. (**c**) NC III and LCE III. (**d**) NC IV and LCE IV.

To locate the mechanical defect, phase-space reconstruction is carried out on the vibration signals of the different vibration substages of the circuit breaker under two operating states, and the chaotic features are extracted. The comparison of the reconstruction results of each vibration substage is shown in Figure 9.

The comparison results of the LLE, MCD, and VDO characteristic values of the overall vibration signal and the signals at each substage extracted from the circuit breaker under two operating states are shown in Table 2. As seen from the table, there is little difference in the characteristic values of the two vibration signals at the overall stage, and it is difficult to distinguish the mechanical states. By comparing the waveform and chaotic features of the vibration signal under each vibration substage, one can find that the features of the two states in substage I are obviously different, and the two states can be distinguished well. Since the circuit breaker closing electromagnet only operates in closing substage I of the circuit breaker, the loosening defect of the closing electromagnet mainly affects the chaotic features of closing vibration signal substage I (the switch release substage). As shown in the phase-space trajectory diagram of each closing substage, the phase-space trajectory of defect state substage I is smaller than that of the normal state, which is caused by the loosening of the electromagnet.

**Figure 10:**
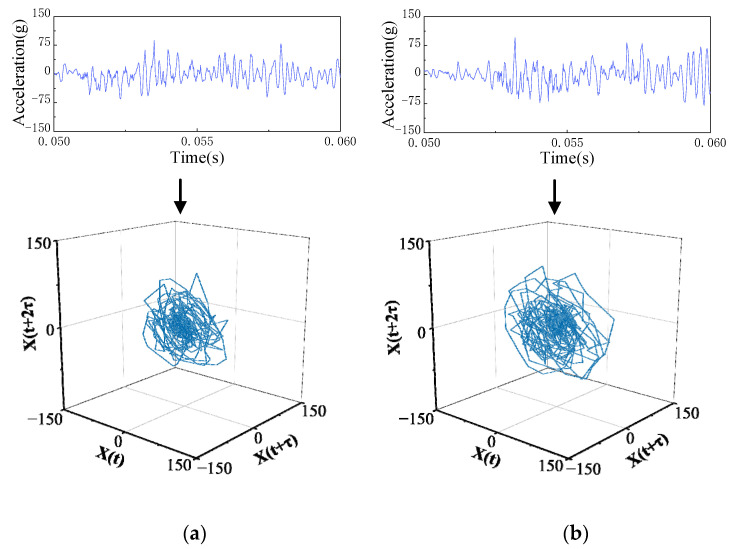
Phase-space reconstruction results of NO and OMJ substage III. (**a**) NO substage III. (**b**) OMJ substage III.

**Figure 11:**
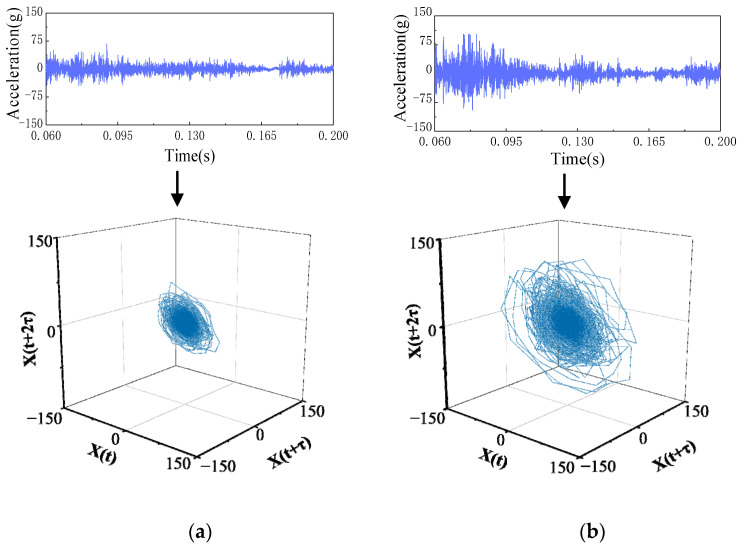
Results of phase-space reconstruction in NO and SAM substage IV. (**a**) NO substage IV. (**b**) SAM substage IV.

Similarly, for the two mechanical defects of the OMJ and SAM, because the defective parts only operate when the circuit breaker is opened, it is only necessary to calculate the LLE of each substage of the opening vibration signal and carry out phase-space reconstruction to extract the features. A comparison of the signal attractor morphology in vibration substage III between the OMJ defect and normal opening of the circuit breaker and a comparison between the SAM defect and the circuit breaker’s normal opening substage IV signal attractor morphologies are shown in Figure 10 and Figure 11. For the OMJ defect, the attractor form of the circuit breaker in vibration substage III is significantly more dispersed than that of the normal state, while for the SAM defect, the attractor form of the circuit breaker in vibration substage IV is larger than that of the normal state.

### 4.3. Mechanical Defect Identification of the Circuit Breaker Operating Mechanism

#### 4.3.1. Mechanical Defect Vibration Substage Localization

**Figure 12:**
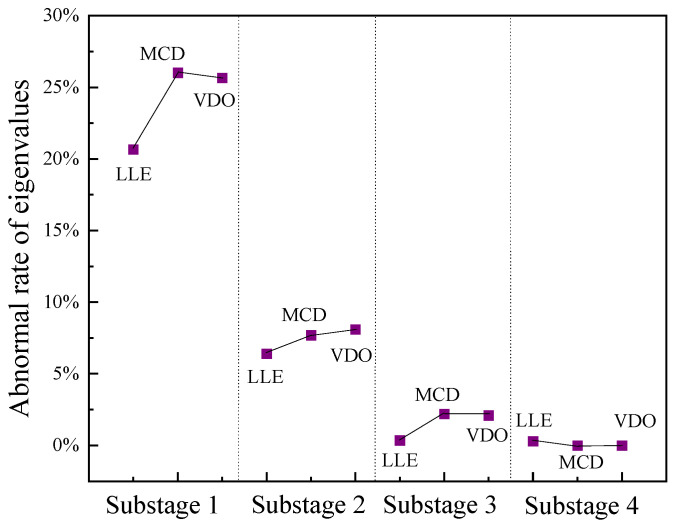
Abnormal rates of characteristic values in each substage of LCE.

For a new signal collected in a practical application, to quantitatively analyze the vibration substage in which the abnormal part of the signal is located, the percentage deviation between the characteristic value of the new signal vibration substage and that of the corresponding vibration substage of the normal signal is calculated, and the abnormal rate of the characteristic value k is defined as follows:

(5) $$k=\frac{\left|T_{e}-N_{e}\right|}{N_{e}}\times 100\%$$

where $T_{e}$ is the characteristic value of the signal to be tested, and $N_{e}$ is the characteristic value of the normal switching signal. For vibration signals of the loosening defect of the closing electromagnet, the abnormal rates of the characteristic values under the four vibration substages are calculated; the results are shown in Figure 12.

The abnormal rates of the three eigenvalues in substage I are all above 20%, while the abnormal rates of the eigenvalues in other substages are all below 10%. In this paper, the threshold value of the abnormal rate is set at 15%. If the abnormal rate of the eigenvalues exceeds 15%, the signal in this substage is considered abnormal and can be used to locate the mechanical defect. In practical engineering applications, this method can be used to calculate the eigenvalue anomaly rate of each vibration substage after the time-sharing division processing of vibration signals to determine the substage where mechanical defects occur and then check the mechanical parts in this substage, so that the mechanically defective parts can be quickly and accurately located.

**Figure 13:**
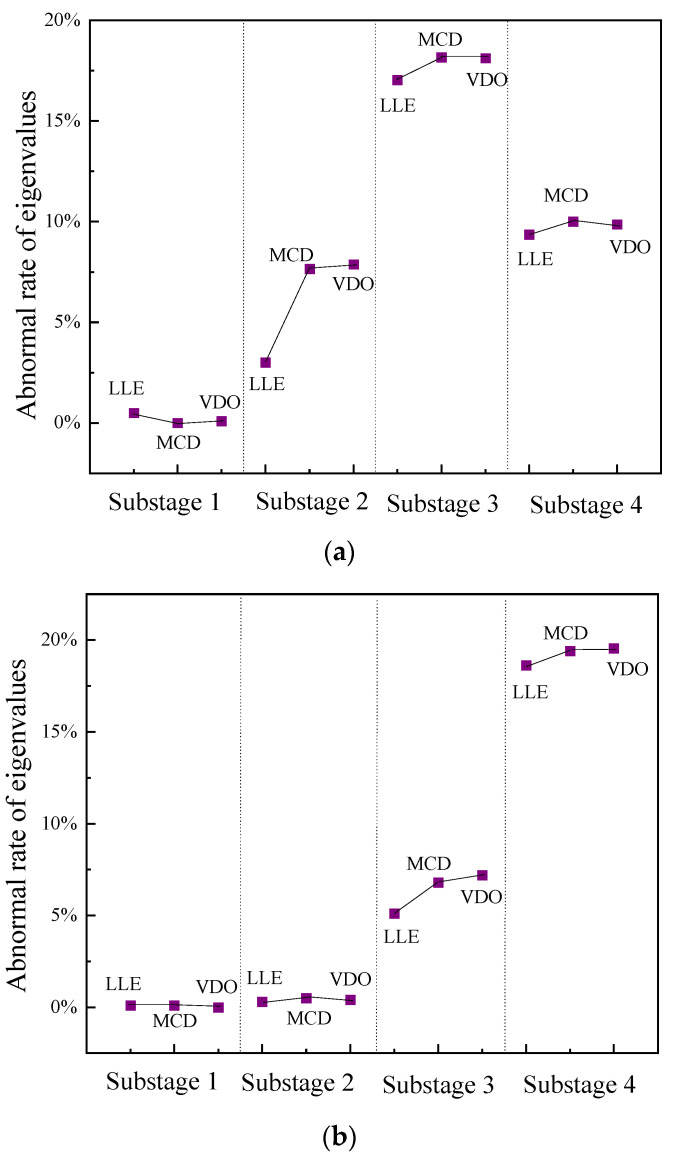
Abnormal rates of eigenvalues at each substage of the fault signal of the opening defect. (**a**) OMJ defect. (**b**) SAM defect.

After extracting the features of the opening signal substages, one finds that the features of the vibration signal under the OMJ defect are significantly different from those of the normal state in vibration substage III (the transmission substage of the mechanism), and the features of vibration substage IV (the braking substage of the mechanism) are significantly different from those of the normal state in the vibration signal of the SAM defect. The abnormal rates of the eigenvalues of each vibration substage under the two operating states were calculated, and the calculated results are shown in Figure 13. As seen from the variation chart of the eigenvalue anomaly rates for the vibration signal under the defect of a jammed operating mechanism, the eigenvalue anomaly rate of vibration substage III is the largest and greater than 15%, while those of the other substages are below the limit value of 15%. Therefore, the mechanical defect occurs in vibration substage III, that is, the transmission substage of the mechanism. For the vibration signal under shock absorber malfunction, only the abnormal rate of the eigenvalue of vibration substage IV is above the limit value; therefore, the mechanical defect may be in vibration substage IV, that is, the mechanism braking substage. The buffer is the main moving part of the braking substage. Therefore, the field staff can quickly locate the abnormal occurrence of the opening buffer according to this method. The jamming defect of the operating mechanism mainly affects the chaotic properties and attractor morphology of opening vibration substage III, while the shock absorber malfunction mainly affects the chaotic properties and attractor morphology of opening vibration substage IV; these results are consistent with the operation characteristics of the mechanical parts of the circuit breaker.

The chaotic features of each substage of the vibration signal are extracted, and the abnormal rates of the eigenvalues of each vibration substage are calculated. The vibration substage of the abnormal signal can be quickly determined, and the mechanical defect can be located according to the actions of the circuit breaker parts.

#### 4.3.2. GA-SVM Classification and Recognition

Before identifying the mechanical state of circuit breakers, the data are first classified according to different operating states. For the circuit breaker fault operation state and normal operation state, the test collected 15 groups of data for each operation state, a total of 75 groups of data; for the data for each operation state, ten groups were selected as the training data and five groups as the test data, a total of 25 groups of test samples, which were used to validate the effects of the classification and recognition. The classification labels are shown in Table 3.

**Figure 14:**
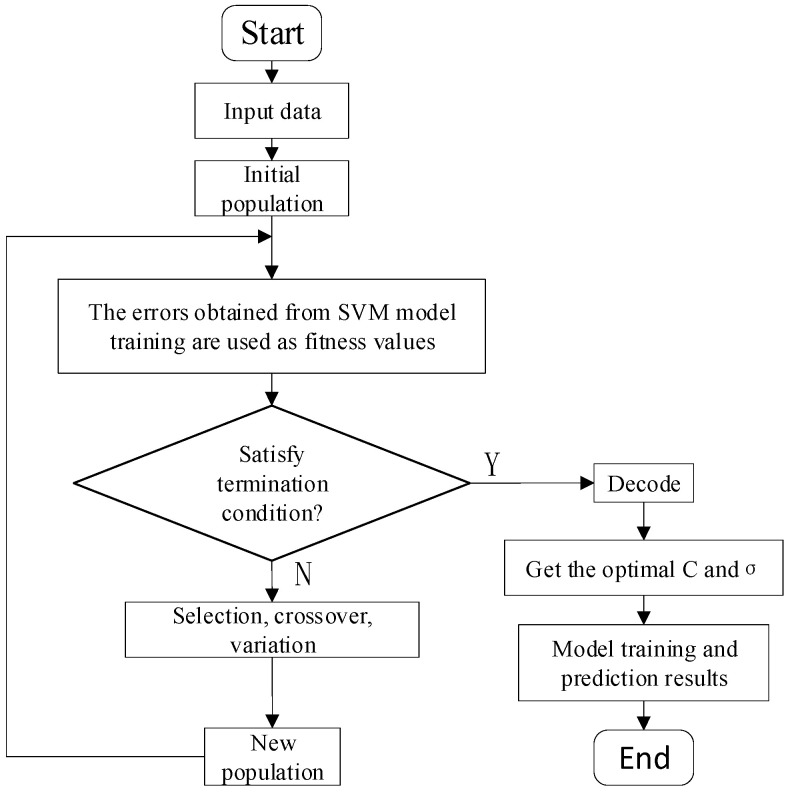
GA-SVM diagnosis model.

To verify that the proposed time-sharing division processing and feature extraction method can effectively distinguish different operating states of circuit breakers, an SVM is used to classify and recognize different types of feature vectors. The amount of vibration signal data collected by the experiment are limited and are classified as a small sample. SVM maps nonlinear test and prediction samples to high-dimensional space through a kernel function and constructs a hyperplane for classification, which is suitable for dealing with the classification of small sample data [37]. The penalty factor c and kernel parameter σ are the most important factors affecting the classification accuracy of SVM. To reduce the error of classification results caused by the selection of parameters c and σ, the parameters c and σ are taken as optimization variables, a genetic algorithm (GA) is selected for parameter optimization, and fitness function values are calculated through population iteration to find the optimal parameters. The initial values of penalty factor c and kernel parameter σ are first randomly generated according to binary coding, the accuracy of SVM classification results as a fitness function and the optimal feature parameters c and σ are searched for by continuous iteration according to the process shown in Figure 14. 

**Figure 15:**
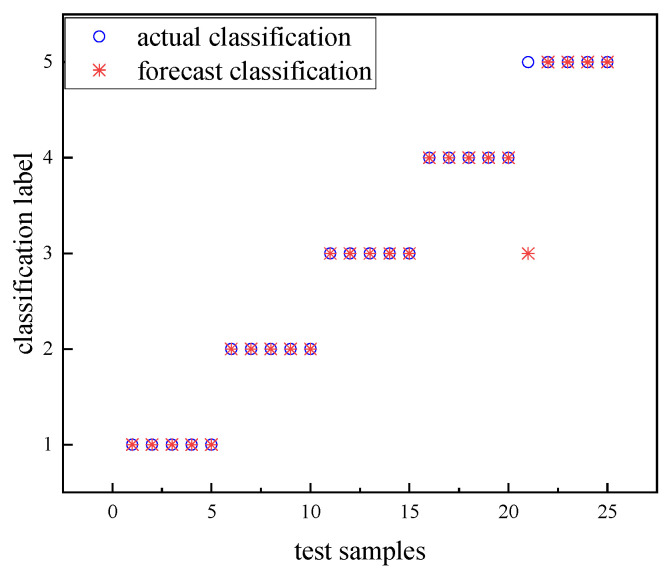
GA-SVM recognition results.

The three eigenvalues of the four substages of the circuit breaker vibration signal are combined to form a one-dimensional eigenvector containing 12 features. The data are labeled according to the defect type, and GA-SVM is used for classification and identification. As the number of iterations increases, the fitness value of the GA gradually increases and converges to a stable value at generation 21, when the parameters are c=1.58 and g=0.1, and the fitness value is 96%. Therefore, after genetic algorithm optimization, the accuracy of the GA-SVM recognition results reached 96%. The GA-SVM recognition results are shown in Figure 15. The identification results show that this method can accurately identify the common mechanical defects of circuit breakers.

The method of time-sharing division of vibration signals proposed in this paper can not only realize the faster and more effective location of mechanical defects, but also has a higher sensitivity to mechanical defects, which can improve the accuracy of fault diagnosis and is more conducive to practical applications in the engineering field.

## 5. Conclusions

This paper studies the vibration signals of HVCBs and proposes a method that divides the interval of the vibration signal and then restructures the phase space of the vibration substage signals to extract chaotic features to locate and identify defects. The study shows the following:

(1) According to the operation characteristics of the spring operating mechanism of the HVCB, the vibration signal of the switch is divided into four substages, namely, the switch release substage, the spring release substage, the mechanism transmission substage, and the mechanism brake substage, by using the method of time-sharing division;
(2) Selecting the LLE, MCD, and VDO as chaotic features to identify the mechanical defect state of the circuit breaker helps achieve a better recognition effect;
(3) Calculating the eigenvalue anomaly rate *k* for each substage of vibration and comparing it to a threshold value of 15%, the vibration substage of the abnormal signal can be quickly determined, and the mechanical defect can be located according to the actions of the circuit breaker parts. Finally, a GA-SVM classifier is used to identify the mechanical defects of circuit breakers with an accuracy of 96%;
(4) The method of time-sharing division of the vibration signal proposed in this paper is suitable for spring-operated circuit breakers. Future work will involve dividing vibration substages according to different types and operation characteristics of circuit breakers in engineering practice.

## Figures and Tables

**Table 1:** LLEs at different substages of the vibration signal under different states. (NC: normal closing, LCE: closing electromagnet looseness, NO: normal opening, SAM: shock absorber malfunction, OMJ: operating mechanism jam).

Status of Operation	Substage I	Substage II	Substage III	Substage IV
NC	0.3051	0.2446	0.2372	0.0447
LCE	0.1900	0.2479	0.2417	0.0551
NO	0.3130	0.2019	0.3666	0.1089
SAM	0.2903	0.1989	0.3642	0.1304
OMJ	0.2977	0.2533	0.3265	0.0978

**Table 2:** Comparison of characteristic values of NC and LCE signals at different substages.

	NC			LCE		
	LLE	MCD	VDO	LLE	MCD	VDO
Whole signal	0.1868	26.7	15.4	0.1837	26.9	15.5
Substage Ⅰ	0.2748	19.6	11.3	0.2180	14.5	8.4
Substage Ⅱ	0.1939	32.3	18.6	0.1815	34.8	20.1
Substage Ⅲ	0.2613	32.4	18.7	0.2604	33.1	19.1
Substage Ⅳ	0.2348	24.1	13.9	0.2355	24.1	13.9

**Table 3:** Classification labels. (NC: normal closing, LCE: closing electromagnet looseness, NO: normal opening, SAM: shock absorber malfunction, OMJ: operating mechanism jam).

Status of Operation	Training Samples	Test Samples	Tag Type
NC	1~10	11~15	1
LCE	16~25	26~30	2
NO	31~40	41~45	3
SAM	46~55	56~60	4
OMJ	61~70	71~75	5

## Data Availability

The data that support the findings of this study are available upon reasonable request from the authors.
